# A Misplaced Right Superior Cuspid Tooth, with Resulting Sequelæ

**Published:** 1888-03

**Authors:** W. W. H. Thackston


ARTICLE III.

### A MISPLACED RIGHT SUPERIOR CUSPID TOOTH, WITH RESULTING SEQUELÆ.

REPORTED BY W. W. H. THACKSTON, M. D., D. D. S.

About the first of September, 1885, Miss Ellen F., aged between thirty-five and forty years, applied for the removal of a few remaining stumps and roots, for the purpose of having inserted an upper set of artificial teeth.

Upon examination I found nothing unusual or abnormal. The lady had previously submitted to the extraction of her teeth as they broke down and became annoying, by her family physician, leaving in her upper jaw only some half dozen stumps and troublesome roots. The sockets and processes of the extracted teeth were well absorbed, the gums shrunken, clean and well rounded, the roof of the mouth, the palate processes of the superior maxillary bones, and the palate bone itself, were all covered with the usual and normal cushion of soft tissue, with no observable ridge, prominence or projection, showing but the normal rugae, and they not noticeably prominent. Neither to the eye nor to the touch was there the slightest indication of the presence of a concealed tooth or the fragment of a root.

In a few months an artificial set was made and inserted. The adaptation, antagonism and arrangement of the teeth was satisfactory in every respect, and for two years the teeth were worn and used with the greatest comfort and enjoyment. I regarded the operation as a "success," for I had several times met my patient and always found her grateful, charmed and enthused with her "new teeth," which she declared were more comfortable, useful and ornamental than her natural ones had ever been.

My surprise and disappointment may be imagined when, a few weeks since, my patient was brought to my office in s state of excitement and nervous prostration that induced a fainting fit soon after taking her seat in my chair, and before I could even make an examinatlon of her trouble. The account given of her case was that a few weeks previously, while visiting friends in the city of Lynchburg, Virginia, she found the roof of her mouth getting tender and painful; the attachment of the plate became insecure; the teeth appeared too long, and did not antagonize the lower ones properly; the pain and swelling in the roof of the mouth increased, until the teeth could not be worn. A dentist was consulted, who said he thought the plate pressed too much upon the "roof," and proceeded to scrape and cut away as much as the thickness of the plate would allow. This afforded no relief; the swelling of tissue progressed, pain and systemic disturbance increased, and, finally, abscess formed, pointed, and discharged freely. The discharge continued; the swelling and enlargement did not subside; the pain was not relieved; the lump, or tumor, became hard and unyielding to pressure. The dentist was again consulted, and he informed the lady that he did not understand her case; feared it was serious; that she had better see a physician, or go to the dentist who had made her plate. A city physician was at once called in, who pronounced the case a grave one; said a bony tumor had developed which might be malignant; that she had better see a surgeon, etc. Miss F. returned in haste to her home and at once called in her family physician, whose examination and diagnoses, unfortunately, confirmed the views of the Lynchburg dentist and physician, and greatly increased her anxiety and apprehensions. The doctor declined to treat the case, and advised the lady to see me without delay.

In a day or two Miss F. was sufficiently recovered from her fainting paroxysm (which, by the way, was one of the most protracted and difficult of management that I ever encountered,) to submit to an examination and any operation determined upon. I had invited and had present my friend, Dr. Vaughan; of Farmville, a skillful and accomplished general surgeon, to assist me in the examination and in whatever operation we might find advisable. I commenced the examination by passing an exploring probe through the sinus which had been formed by the discharge of pus and ichor, which sinus opened a little below and just back of the foramen incisivum, leading back along the median line in the direction of the palate bone. I was at once rewarded by a sharp, ringing sound, and that impression of touch with which dentists are so familiar. I found the point of resistance to be a little roughened at its anterior extremity. Continuing the exploration, I found the shape of the hard body to be oval and oblong. Without remark I handed the probe to Dr. Vaughan, who repeated the examination, and, in doing so, he struck the roughened surface to which I have alluded. The doctor, being a physician and general surgeon, and of course not familiar with what we designate "the enamel touch" when we came to consult and compare notes, was inclined to question my diagnosis, that it was simply a misplaced and imbedded tooth, with an enameled crown and fully developed root. We, however, agreed that whether a tooth or a bony growth, its prompt removal was indicated. Our patient being now reassured and in good spirits, readily consented that the operation might at once be proceeded with, and bravely declined the use of any anæsthetic.

Feeling very confident that my diagnosis of the case would be verified, I determined to first look and feel for a tooth, and, if successful, at once remove it. With a simple curved gum-lancet I made an incision down to the enlargement, a little more than half an inch, in length, and at the most dependent and prominent point, a transverse incision. I then dissented around the hard substance as far as practicable and introduced a very narrow, thin-bladed root-forceps, with which, after some difficulty, I succeeded in getting a firm hold; gently feeling my way, and cautiously applying force, I recognized some motion and yielding in the object embraced by the forceps, satisfying me that there was slight, if any, bony adhesion. I at once applied about the force usually employed in extracting a bicuspid tooth, disengaged and took away a fully developed cuspid an inch in length, crown and root, with none of the characteristics of a supernumerary, but just such a tooth in size and development as should have been found in its natural position in the dental arch. The tooth lay alongside and parallel with the palatine suture, a little to the right of the median line, and was attached to the palate process by its inner or lingual surface, the crown and cusp pointing forward and the root running back nearly or quite to the palate bone. There was no root socket or alveolar matrix. The attachment, though firm and strong, was simply fibrous and periosteal. The enamel had been eroded and roughened, I think, by the acrid secretion of the abscess, and the point of the cusp had either been chemically dissolved or, possibly, nibbled away by the bugs—the "microbes." It was that roughness which mystified us in the examination, as we had to rely solely upon the "touch."

The recovery was rapid and complete. A little uneasiness was felt for a day or two in the right maxillary sinus and nostril, but in three days the teeth were again in the mouth, the plate fitting as perfectly as when first inserted, and the denture rendering comfortable and satisfactory service. A month had elapsed, and no trace or symptom of the trouble remains.

We can readily understand how an unerupted tooth could occasion all the disturbance, suffering, doubt and uncertainty as to the cause and true character of the manifestations and expressions in this case. We can understand very readily how dentists, physicians and surgeons could be mystified and misled in making a diagnosis, but the perplexing and embarrassing question is, where did that tooth come from? Where was it when I made a careful examination and first took the impression for the artificial denture?

It was certainly not at that time in the roof of the mouth, and there was no appearance or indication of the presence of a tooth or root, or fragment of a stump or root, in or about the upper jaw. I do not think it reasonable or probable that a straggling or wandering follicle or tooth germ should have fixed itself upon the palate process, and in two years have developed such a tooth; and even if so, I should have expected the trouble to have been gradually manifested, a slow but progressive growth, and increasing uneasiness; but in this case the manifestations were sudden —a period of a few weeks comprised the origin, progress and treatment of the case.

There was no opening (that could be discovercd) into the maxillary sinus, but I think the tooth grew and developed, in some niche or recess in the irregular walls of the antrum, and from some cause, possibly the application of a plate holding a set of teeth, there was excited and stimulated a change of position, and the tooth found its way to the point from which it was taken. This, however, is mere hypothesis and conjecture. Of what I am assured and certain in the case may be briefly summed up. There was no tooth in the roof of the mouth when I inserted the artificial teeth; for nearly or quite two years the plate was worn with comfort, suddenly, and without premonition, the trouble came on, with the symptoms and conditions described, and perfect relief and cure was accomplished by finding and removing the hidden tooth, the source and cause of great suffering and mental distress to the patient and great perplexity to the professional gentlemen who were consulted in the case.

I trust the readers of the *Journal* will excuse this lengthy and circumstantial report, when I assure them it has been written solely for the benefit of the profession, especially the younger members. It may be their fortune to encounter such a case, and they need not always anticipate, or diagnose *exostosis*, *bony nodules* or *osteo sarcomas*.

[The tooth, Dr. Thackston sent us for examination. It would not be taken for a supernumerary tooth, as it is of good shape and length.—ED.]—*Southern Dental Journal.*

---

ARTICLE IV.

### AMMONIA VS. ZINC FILLINGS.

